# Yap drives the development of cardiovascular disease in patients with rheumatoid arthritis

**DOI:** 10.5937/jomb0-45932

**Published:** 2024-04-23

**Authors:** Guozhu Che, Ying Liu, Na Zhang, Jing Zhao

**Affiliations:** 1 Shanxi Bethune Hospital, Department of Rheumatology and Immunology, Shanxi Academy of Medical Sciences, Tongji Shanxi Hospital, Third Hospital of Shanxi Medical University, Taiyu, China

**Keywords:** rheumatoid arthritis, cardiovascular disease, YAP, reumatoidni artritis, kardiovaskularne bolesti, YAP

## Abstract

**Background:**

To assess the influence of serum level of YAP on laboratory examination findings, imaging findings and disease activity of rheumatoid arthritis patients combined cardiovascular disease (RA-CVD).

**Methods:**

RA-CVD patients (n=60), RA-nCVD patients (n=60) and healthy subjects (n=60) were recruited. Serum levels of YAP in them were detected by qRT-PCR. Their baseline characteristics were analyzed and compared. Disease activity, CVD risk factors and imaging findings in RA-CVD and RA-nCVD patients were evaluated and compared. In addition, potential influences of YAP on disease activity, CVD risk factors and imaging findings in RA-CVD patients were assessed.

**Results:**

RA-CVD patients had higher levels of ERS, anti-CCP, RF, HDL-C, CRP, FRS, BNP, LA, LVs, LVd and cIMT, and lower level of EF in comparison to RA-nCVD patients. Serum level of YAP was higher in RA-CVD patients than that of RA-nCVD patients and healthy subjects. YAP level was positively correlated to DAS28, TG, CRP, PLT, FRS, BNP and cIMT in RA-CVD patients.

**Conclusions:**

Serum level of YAP increases in RA-CVD patients. YAP is a potential factor driving the development of CVD in RA patients through regulating inflammatory response, lipid metabolism, glycometabolism and thrombosis.

## Abstract (sr)

**Uvod:**

Ispitivana je procena uticaja serumskog nivoa YAP na nalaze laboratorijskih pregleda, nalaz imidžinga i aktivnost bolesti kod pacijenata sa kombinovanim kardiovaskularnim oboljenjem sa reumatoidnim artritisom (RA-CVD).

**Metode:**

Regrutovani su pacijenti sa RA-CVD (n=60), pacijenti sa RA-nCVD (n=60) i zdravi ispitanici (n=60). Nivoi YAP u serumu kod njih su detektovani kRT-PCR. Njihove osnovne karakteristike su analizirane i upoređene. Procenjeni su i upoređeni aktivnosti bolesti, faktori rizika za KVB i nalazi imidžinga kod pacijenata sa RA-CVD i RAnCVD. Pored toga, procenjeni su potencijalni uticaji YAP na aktivnost bolesti, faktore rizika za KVB i nalaze imidžinga kod pacijenata sa RA-KVB.

**Rezultati:**

Pacijenti sa RA-CVD su imali viši nivo ERS, anti-CCP, RF, HDL-C, CRP, FRS, BNP, LA, LVs, LVd i cIMT i niži nivo EF u poređenju sa pacijentima sa RA-nCVD. Nivo YAP u serumu bio je viši kod pacijenata sa RA-CVD nego kod pacijenata sa RA-nCVD i zdravih subjekata. Nivo YAP je bio u pozitivnoj korelaciji sa DAS28, TG, CRP, PLT, FRS, BNP i cIMT kod pacijenata sa RA-KVB.

**Zaključak:**

Nivo YAP u serumu raste kod pacijenata sa RA-KVB. YAP je potencijalni faktor koji pokreće razvoj KVB kod pacijenata sa RA kroz regulisanje inflamatornog odgovora, metabolizma lipida, glikometabolizma i tromboze.

## Introduction

Rheumatoid arthritis (RA) is a persistent autoimmune and inflammatory disease characterized by synovial cell proliferation and symmetrical, aggressive inflammation of small joints. RA-induced persistent joint synovitis and damages of joint soft tissues like cartilage and ligaments, eventually leading to bone erosion [1].

There are many extra-articular clinical manifestations of RA, involving cardiovascular system, respiratory system, digestive system, nervous system and skin. Notably, the cardiovascular system is the most affected during the active phase of RA, which is the major reason leading to RA death (>50%) [2]. It is reported that RA patients develop CVD earlier than healthy people, and the incidence of a sudden cardiac death in the former group is twice that of the latter [3]. In addition, incidences of secondary myocardial infarction, asymptomatic heart failure, ischemic cardiomyopathy and sudden death in RA patients are twice that of healthy people [4].

Yes-associated protein (YAP) is a multifunctional intracellular connexin and transcription coactivator [5]. Recent studies have shown the vital function of YAP in the development of cardiovascular system. Del Re et al. [6] discovered cardiomyocyte apoptosis and fibrosis, dilated cardiomyopathy and premature death of mice with cardiomyocyte-specific homozygous inactivation of Yap1. In addition, YAP-inactivated mice also present impaired cardiac regeneration because of the absence of fibrotic response. Activated YAP stimulates the regeneration of myocardial cells and enhances the contractility of myocardium, thus improving heart function [7] [8]. YAP is reported as a vital gene in the development of RA [9]. This study aims to explore the influence of YAP on the development of RA-CVD by retrospectively analyzing their clinical data.

## Materials and methods

### Subjects

RA-CVD patients (n=60) and RA-nCVD patients (n=60) were hospitalized in Shanxi Bethune Hospital from 2017 to 2023. They were confirmed as RA according to the American Rheumatic Association (ACR) 1987 revised criteria or the European League Against Rheumatism (EULAR) 2010 guidelines. CVD mainly included: (1) Myocardial ischemia, myocardial infarction, ventricular high voltage and arrhythmia detected by electrocardiogram; (2) Heart valve disease, pericarditis, pericardial effusion and myocardial disease detected by echocardiography; (3) Carotid intima thickening, atherosclerosis, or plaque formation detected by color ultrasound of carotid artery [10] [11]. The following subjects were excluded from this trial: (1) Patients with other autoimmune or connective tissue diseases (e.g. rheumatic fever, idiopathic inflammatory myopathy, systemic lupus erythematosus, ANCA related vasculitis, ankylosing spondylitis, psoriatic arthritis); (2) Patients with specific or non-specific infection, diabetes or tumors; (3) Patients with rheumatic valvular disease, senile degenerative valvular disease and other diseases related heart damage. A total of 60 healthy subjects received physical examinations during the same period were recruited as controls. This study conformed to the ethical standards, and was approved by the hospital ethics committee and informed consent of subjects.

### Clinical data collection

Disease activity indicators, CVD risk factors and imaging findings of RA-CVD and RA-nCVD patients were recorded as follows: (1) Disease activity: DAS28 (disease activity for 28 joint indices score), RF (rheumatoid factor), anti-CCP (anti-cyclic peptide containing citrulline) and ESR (erythrocyte sedimentation rate); (2) CVD risk factors: TC (total cholesterol), TG (triglycerides), HDL-C (high-density lipoprotein cholesterol), CRP (c-reactive protein), PLT (platelet), BNP (brain natriuretic peptide), CK (creatine kinase), CK-MB (creatine kinase isoenzyme), FRS (Framingham risk score), D-D polymer and cTnI (troponin I); (3) Imaging findings: EF (ejection fraction), LA (left atrial diameter), LVd (left ventricular diameter diastolic period), LVs (left ventricular diameter systolic period) and RV (right ventricular diameter).

### Disease activity evaluation of RA

DAS28 was used to evaluate disease activity of RA, including 28 joints as follows: bilateral shoulders, elbows, wrists, interphalangeal joints and knees. Number of joints with tenderness upon touching (TEN28) and swelling (SW28), ESR, CRP and the subjective assessment (SA) were taken into the calculation of DAS28 [11]: DAS28= 0.56 × + 0.28 × + 0.70 × ln (ESR) + 0.014 × SA.

### Echocardiography

Transthoracic echocardiography was conducted in each subject. The subject was maintained at a left lateral position for recording LVs, LVd, RV, LAs and EF.

### Color ultrasound of carotid artery

CIMT was measured using the Logiq E9 (intermediate frequency 9 MHz) at three locations: Common carotid artery, bifurcation and internal carotid artery. CIMT >1.0 mm and >1.3 mm indicated intimal thickening and atherosclerotic plaque, respectively.

### qRT-PCR

Serum RNA was isolated using TRIzol, and reversely transcribed to cDNA using AMV reverse transcription kit (TaKaRa, Tokyo, Japan). Using the cDNA as the template, qRT-PCR was conducted to detect YAP level. Primer sequences were: YAP: 5'-CGCTCTTCAACGCCGTCA-3' (forward) and 5'-AGTACTGGCCTGTCGGGAGT-3' (reverse); GAPDH: 5'-CTCCTCCTGTTOGACAGTCAGC-3' (forward) and 5'-CCCAATACGACCAAATCCGTT-3' (reverse).

### Statistical process

Statistic Package for Social Science (SPSS) 22.0 (IBM, Armonk, NY, USA) was used for statistical processing. Measurement data were expressed as mean±S.D. Differences between groups and among multiple groups were analyzed by the t test and one-way ANOVA, respectively. Enumeration data were expressed as percentage and compared by Chi-square test. Pearson correlation test was conducted to assess the correlation between YAP level and disease activity, CVD risk factors and imaging findings in RA-CVD patients. *P*<0.05 considered as statistically significant.

## Results

### Baseline characteristics of subjects

RA-CVD patients (n=60), RA-nCVD patients (n=60) and healthy subjects (n=60) were recruited in this trial. In detail, there were 27 men and 33 women with an average age of 55.2±8.3 years in RA-CVD group. RA-nCVD group had 24 men and 36 women with an average age of 56.3±8.9 years. In healthy control group, there were 26 men and 34 women, and their average age was 55.7±8.1 years. Age and sex rate were comparable among the three groups (Table 1).

**Table 1:** Baseline demographics of subjects.

Variable	RA-nCVD<br>(n=60)	RA-CVD<br>(n=60)	Health control<br>(n=60)	t/χ^2^	P
sex<br>(male/female)	24/36	27/33	26/34	0.318	0.853
age<br>(years old)	56.3±8.9	55.2±8.3	55.7±8.1	2.253	0.108

### Disease activity in RA-nCVD and RA-CVD patients

In comparison to RA-nCVD patients, RA-CVD patients had higher ERS (61.42±20.74 mm/h), anti-CCP (169.34±57.41 μmol/L) and RF (157.84±73.82 U/mL) (*P*<0.05). However, no significant difference was detected in DSA28 between groups (*P*>0.05) (Table 2). It is indicated that ERS, anti-CCP and RF wound affect the occurrence of CVD in RA patients.

**Table 2:** Analysis of disease activity between RA-nCVD group and RA-CVD group. Note: ESR: Erythrocyte Sedimentation Rate; DAS28: Disease Activity for 28 joint indices score; Anti-CCP: Anti-Cyclic Citrullinated Peptide antibodies; RF: Rheumatoid Factor

Variable	RA-nCVD<br>(n=60)	RA-CVD<br>(n=60)	t	P
ESR<br>(mm/h)	45.28±14.33	61.42±20.74	-4.959	<0.001
DAS28	4.12±0.58	4.38±0.96	-1.796	0.075
Anti-CCP<br>(mmol/L)	48.58±11.59	169.34±57.41	-15.971	<0.001
RF (U/mL)	100.58±48.32	157.84±73.82	-5.027	<0.001

### Cardiovascular risks in RA-nCVD and RA-CVD patients

Among traditional cardiovascular risks, higher level of HDL-C was detected in RA-CVD patients than that of RA-nCVD patients (*P*<0.05). We did not detect significant differences in TC and TG between groups (*P*>0.05). By analyzing non-traditional cardiovascular risks, it is found that CRP (48.33±13.53 mg/dL), FRS (14.86±5.83) and BNP (378.48±183.63 pg/mL) were much higher in RA-CVD patients compared with RA-nCVD patients (*P*<0.05). PLT, D-D polymer, CK, CK-MB and cTnI were comparable between groups (*P*>0.05) (Table 3).

**Table 3:** Analysis of cardiovascular risk factors between RA-nCVD group and RA-CVD group. Note: TC: Total Cholesterol; TG: Triglycerides; HDL-C: High-Density Lipoprotein Cholesterol; CRP: C-reactive protein; PLT: platelet; CK: Creatine Kinase; cTnI: Troponin I; FRS: Framingham Risk Score; BNP: Brain Natriuretic Peptide

Variable	RA-nCVD	RA-CVD	t	P
TC (mmol/L)	4.29±1.04	3.97±0.88	1.819	0.071
TG (mmol/L)	3.94±1.23	4.33±1.35	-1.654	0.101
HDL-C (mmol/L)	0.88±0.38	1.27±0.82	-3.343	<0.001
CRP (mg/dL)	36.35±9.54	48.33±13.53	-5.605	<0.001
PLT (*109/L)	214.68±63.41	203.84±60.74	0.956	0.341
D-D polymer (mg/mL)	2.89±0.79	2.94±0.84	-0.336	0.738
CK (U/L)	35.98±7.58	34.61±7.11	1.021	0.309
Ck-MB (U/L)	16.03±6.81	15.83±6.52	0.164	0.870
cTnI (ng/mL)	0.37±0.22	0.36±0.22	0.249	0.804
FRS	10.37±5.34	14.86±5.83	-4.588	<0.001
BNP (pg/mL)	37.48±18.48	378.48±183.63	-14.312	<0.001

### Imaging findings in RA-nCVD and RA-CVD patients

The length of stay of RA-CVD patients was longer than that of RA-nCVD patients, and part of them had atrial or ventricular hypertrophy. As a result, LA (39.47±3.88 mm), LVs (34.76±4.04 mm) and LVd (48.49±5.15 mm) were larger in RA-CVD patients than those of RA-nCVD patients. EF (45.83±7.04%) was lower in RA-CVD patients. A larger cIMT was detected in RA-CVD patients (1.18±0.44 mm) compared with RA-nCVD patients (P<0.05) (Table 4).

**Table 4:** Analysis of ultrasonography between RA-nCVD group and RA-CVD group. Note: LA: Left Atfial diameter; LVs: Left Ventricular diameter Systolic period; LVd: Left Ventricular diameter Diastolic period; RV: Right Ventricular diameter; EF: Eiection Fraction; cIMT: carotid Intima Media Thickness

Variable	RA-nCVD<br>(n=60)	RA-CVD<br>(n=60)	t	P
LA (mm)	35.67±3.45	39.47±3.88	-5.669	<0.001
LVs (mm)	30.25±3.78	34.76±4.04	-6.314	<0.001
LVd (mm)	43.97±4.86	48.49±5.15	-4.944	<0.001
RV (mm)	19.76±2.45	19.01±1.81	1.907	0.059
EF (%)	59.34±7.93	45.83±7.04	9.869	<0.001
cIMT (mm)	0.89±0.31	1.18±0.44	-4.173	<0.001

### Serum level of YAP

**Figure 1:**
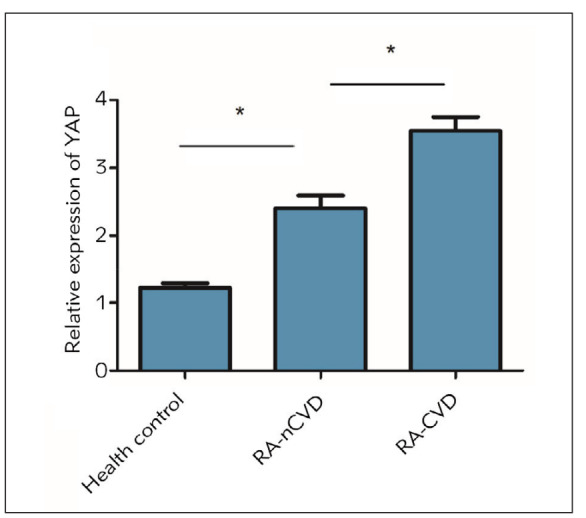
Serum level of YAP. Serum levels of YAP in healthy subjects, RA-nCVD patients and RA-CVD patients.

Serum level of YAP was much higher in RA-CVD patients compared with that of healthy subjects and RA-nCVD patients. Moreover, RA-nCVD patients had higher serum level of YAP than healthy subjects (*P*<0.05) (Figure 1). It is speculated that YAP may be a risk factor triggering the development of RA-CVD.

### Correlation between YAP and disease activity of RA-CVD

Pearson correlation test was conducted to assess the correlation between YAP and disease activity of RA-CVD. It is shown that serum level of YAP was positively correlated to DAS28 in RA-CVD patients (r=0.321, *P*<0.05). However, it had no correlation to RF, anti-CCP and ESR in RA-CVD patients (Table 5).

**Table 5:** Correlation of YAP expression level from Serum of RA-CVD patients with Disease Activity. Note: ESR: Erythrocyte Sedimentation Rate; DAS28: Disease Activity for 28 joint indices score; Anti-CCP: Anti-Cyclic Citrullinated Peptide antibodies; RF: Rheumatoid Factor

Variable	r	P
ESR (mm/h)	-0.183	0.753
DAS28	0.321	0.006
Anti-CCP (mmol/L)	0.385	0.105
RF (U/mL)	-0.151	0.083

### Correlation between YAP and cardiovascular risks of RA-CVD

Among traditional cardiovascular risks, serum level of YAP was only positively correlated to TG (r=0.532, *P*=0.004), while it had no correlation to TC and HDL-C in RA-CVD patients. Among non-traditional cardiovascular risks, YAP level was positively correlated to CRP, PLT, FRS and BNP (r=0.633, 0.731, 0.485 and 0.773, respectively, *P*<0.05) in RA-CVD patients. No significant correlation was discovered between YAP and D-D polymer, CK, CK-MB and cTnI (Table 6).

**Table 6:** Correlation of YAP expression level from Serum of RA-CVD patients with cardiovascular risk factors. Note: TC: Total Cholesterol; TG: Triglycerides; HDL-C: High-Density Lipoprotein Cholesterol; CRP: C-reactive protein; PLT: platelet; CK: Creatine Kinase; cTnI: Troponin I; FRS: Framingham Risk Score; BNP: Brain Natriuretic Peptide

Variable	r	P
TC (mmol/L)	0.321	0.226
TG (mmol/L)	0.532	0.004
HDL-C (mmol/L)	-0.437	0.651
CRP (mg/dL)	0.633	0.009
PLT (*10^9^/L)	0.731	<0.001
D-D polymer	0.164	0.539
CK (U/L)	-0.096	0.375
Ck-MB (U/L)	-0.175	0.446
cTnI (ng/mL)	0.653	0.226
FRS	0.485	0.018
BNP (pg/mL)	0.773	<0.001

### Correlation between YAP and imaging findings of RA-CVD

According to the imaging findings, only cIMT was detected to be positively correlated to serum level of YAP in RA-CVD patients (r=0.435, *P*<0.001). We did not yield the correlation between YAP and LA, LVs, LVd, RV and EF (Table 7).

**Table 7:** Correlation of YAP expression level from Serum of RA-CVD patients with ultrasonography. Note: LA: Left Atfial diameter; LVs: Left Ventricular diameter Systolic period; LVd: Left Ventricular diameter Diastolic period; RV: Right Ventricular diameter; EF: Eiection Fraction; cIMT: carotid Intima Media Thickness

Variable	r	P
LA (mm)	0.442	0.146
LVs (mm)	0.119	0.341
LVd (mm)	-0.324	0.551
RV (mm)	0.421	0.115
EF (%)	-0.355	0.083
cIMT (mm)	0.435	<0.001

## Discussion

YAP is the main downstream effector of the Hippo signaling. In 1995, Sudol et al. [12] identified and cloned a novel protein binding to the SH3 domain of the YES kinase. YAP locates on human chromosome 11q22, containing 2 displacement shears, that is, YAP1 and YAP2. YAP1 contains a WW domain, while YAP2 has two. YAP protein is widely expressed in diverse tissues except peripheral blood leukocytes. YAP has multiple domains or specific amino acid sequences, including the proline-rich domain at N-terminal, TEADs binding region, two WW domains, one SH3 binding motif, transcription activation domain at C-terminal, and PDZ binding motif. Through the unique domains or sequences, YAP is capable of interacting with proteins and thus exerts its diverse biological functions [13] [14].

Research on the role of YAP in CVD has become a hot spot. YAP is important in the development of the heart, which is a key regulator of cardiomyocyte proliferation, cardiac morphogenesis and myocardial trabecular formation [15]. The continuous activation of YAP in the heart of mouse embryos stimulates cardiomyocytes to proliferate and enlarges heart size [15]. Mice with specific knockout of YAP present defects of heart and vascular development [16]. *In vitro* knockdown of YAP induces the expression of cell cycle arrest-associated gene Gpr132, which blocks cells in the G0/G1 phase and thus causes angiodysplasia. Wang et al. [17] found that YAP is upregulated during the phenotypic transformation of smooth muscle cells induced by arterial injury, which further downregulates smooth muscle-specific genes and drives proliferative and migratory functions. A recent study reported that knockdown of YAP reduces pathogenic behaviors of solute fibroblast-like synoviocytes in RA patients and the severity of arthritis, suggesting that YAP is a critical regulator of RA [9]. Our findings uncovered that serum level of YAP was remarkably elevated in RA-CVD patients, showing a potential to promote the occurrence of CVD in RA patients.

The emergence of cytokine targeted therapy confirms the role of cytokines and inflammation in the pathogenesis of RA. High-level inflammation markers, such as CRP, are positively correlated to vascular endothelial dysfunction and impaired vasodilation [18]. RA and other systemic inflammatory diseases are associated with dyslipidemia, characterized by high level of TC, and low levels of LDL and HDL. Although lipoprotein levels are very low, the risk of CVD controversially enhances. The theory of lipid paradox believes that low lipoprotein level is associated with inflammatory response, and the latter is independently linked to CVD. Low lipoprotein level is also linked to ESR and CRP elevation [19]. Lertnawapan et al. [20] detected that serum cystatin C concentration increases in RA-CVD patients, which is positively correlated to inflammation indicators and DAS28. Consistently, our findings showed that serum level of YAP in RA-CVD patients was positively correlated to DAS28, as well as CRP, PLT, FRS and BNP, indicating that YAP could predict the state of inflammatory response and the occurrence of CVD in RA patients. We did not obtain a correlation between YAP level and internal diameters of atria or ventricle in RA-CVD patients. Nevertheless, a positive correlation was identified between YAP and cIMT, which was consistent with previous findings that coronary flow reserve decreases and carotid intima-media thickness increases in RA patients [21].

However, this study also has certain limitations. Firstly, the sample size of patients included in the study is relatively small. Secondly, due to constraints on clinical patient data, we did not perform further statistical analysis on basic patient information such as height and weight, which could potentially introduce some bias into the research results. Finally, although we have provided preliminary evidence through the collection of clinical data that suggests a correlation between YAP and the occurrence and development of RA-CVD, we have not conducted further experiments to demonstrate the specific role of YAP in the onset and progression of RA-CVD. Therefore, in our future research plans, we will focus on exploring the specific mechanisms of YAP in the occurrence and development of RA-CVD. This will contribute to the development of relevant drugs for the prevention and treatment of RA-CVD.

In summary, our study found that serum level of YAP was positively correlated with disease activity, CVD risk factors and other factors in RA-CVD patients. Our conclusion provides a basis for the treatment and pathogenesis analysis of RA-CVD.

## Conclusion

Serum level of YAP of RA patients is positively correlated with the risk of RA-CVD. It may affect the occurrence of RA-CVD by regulating inflammation, lipid metabolism, glycometabolism and thrombosis in RA patients, suggesting that YAP is a potential risk factor of RA-CVD.

## Dodatak

### Conflict of interest statement

All the authors declare that they have no conflict of interest in this work.
